# Supplementary material for: The Effect of Zoledronic Acid on Serum Biomarkers among Patients with Chronic Low Back Pain and Modic Changes in Lumbar Magnetic Resonance Imaging
Source: Diagnostics (Basel). 2019 Dec 4;9(4):212. doi: 10.3390/diagnostics9040212 (PMC6963270; doi:10.3390/diagnostics9040212)
Supplement: Supplementary file 1 [file diagnostics-09-00212-s001.zip › Suppl tables.pdf]

Table S1. Median concentrations and interquartile range (IQR) of serum biomarkers at baseline, one month and one year according to type of Modic changes (M1-dominant (M1) or M2-dominant (M2) P^1 shows the significance of the change in each respective biomarker concentration from baseline to one month or from baseline to one year, separately for M1 and M2 lesions. P^2 shows the significance of the difference in change from baseline to one month or from baseline to one year in the concentration of each biomarker between the patients with M1 and M2 types. Significant *p*-values are bolded.

Serum Biomarkers	Baseline Median (IQR)	1 Month Median (IQR)	P^1	P^2	1 Year Median (IQR)	P^1	P^2
**Bone panel**							
AFOS (U/L)				0.080			**0.003**
M1	67 (59, 79)	65 (57, 76)	0.386		56 (53, 66)	**0.001**	
M2	64 (51, 85)	71 (60, 87)	0.142		67 (54, 91)	0.403	
RANKL (pg/mL)				**0.041**			0.522
M1	9.5 (0.0, 37.0)	0.0 (0.0, 42.8)	0.369		9.5 (0.0, 45.6)	0.498	
M2	19.2 (0.0, 66.9)	19.2 (6.5, 87.1)	0.084		19.2 (0.0, 57.8)	0.672	
iPINP (ng/mL)				**0.014**			**0.003**
M1	38 (27, 50)	30 (21, 40)	**0.001**		18 (14, 35)	**<0.001**	
M2	33 (25, 39)	36 (24, 41)	0.787		37 (18, 44)	0.685	
CTX-1 (pg/mL)				>0.999			0.732
M1	0.3 (0.2, 0.5)	0.1 (0.0, 3.4)	0.515		0.2 (0.1, 4.7)	0.714	
M2	0.2 (0.1, 0.4)	0.1 (0.0, 0.3)	**0.027**		0.2 (0.1, 0.3)	0.893	
**Chemokine panel**							
Eotaxin-1 (pg/mL)				0.197			0.159
M1	178 (147, 256)	191 (140, 241)	0.594		179 (143, 233)	0.485	
M2	170 (139, 252)	197 (153, 218)	0.305		190 (169, 244)	0.305	
Eotaxin-3 (pg/mL)				0.331			0.441
M1	19.5 (14.8, 26.2)	16.4 (11.8, 28.1)	**0.034**		17.7 (10.9, 27.0)	0.155	
M2	17.0 (10.4, 23.2)	17.9 (9.2, 20.8)	0.588		14.3 (12.5, 22.1)	> 0.999	
IP-10 (pg/mL)				0.493			**0.025**
M1	247 (188, 297)	248 (205, 296)	0.129		265 (209, 372)	**0.044**	
M2	303 (212, 464)	356 (213, 446)	0.893		279 (205, 371)	0.168	
MIP-1A (pg/mL)				0.227			0.728
M1	5.3 (5.3, 20.0)	5.3 (5.3, 17.1)	0.639		11.6 (5.3, 17.7)	0.602	
M2	5.3 (5.3, 20.1)	18.4 (5.3, 21.0)	0.557		10.7 (5.3, 19.0)	0.910	
MIP-1B (pg/mL)				0.100			0.512
M1	110 (68, 154)	96 (66, 133)	**0.036**		99 (66, 157)	0.578	
M2	115 (73, 154)	122 (89, 156)	0.588		114 (77, 169)	0.735	
MCP-1 (pg/mL)				0.151			0.303
M1	256 (213, 298)	256 (208, 308)	0.427		267 (209, 323)	0.804	
M2	245 (216, 335)	293 (245, 356)	0.168		300 (230, 345)	0.191	
MCP-4 (pg/mL)				**0.012**			**0.031**
M1	148 (109, 179)	132 (110, 164)	**0.023**		132 (105, 176)	0.413	
M2	122 (105, 177)	148 (129, 208)	0.094		156 (122, 210)	0.080	
MDC-1 (ng/mL)				0.094			0.073
M1	1.1 (0.9, 1.3)	1.0 (0.9, 1.2)	0.348		1.0 (0.8, 1.2)	0.123	
M2	1.0 (0.7, 1.4)	1.0 (0.8, 1.6)	**0.048**		1.1 (0.7, 1.6)	0.305	
RANTES (ng/mL)				0.113			0.197
M1	102 (59, 218)	85 (43, 134)	**0.010**		84 (61, 143)	0.117	
M2	69 (34, 88)	76 (48, 104)	0.893		75 (62, 92)	0.685	
TARC (pg/mL)				**0.007**			**0.004**
M1	295 (180, 432)	270 (168, 369)	0.269		288 (170, 389)	0.427	
M2	267 (153, 367)	290 (235, 466)	**0.013**		337 (214, 449)	0.002	
MIG-1 (pg/mL)				0.983			0.051
M1	72 (28, 119)	83 (28, 119)	0.924		72 (47, 118)	0.090	
M2	72 (56, 110)	72 (65, 162)	0.910		72 (23, 101)	0.156	
**Cytokine panel**							
IL-7 (pg/mL)				0.240			0.690
M1	18.6 (15.2, 22.8)	15.9 (11.7, 19.7)	0.055		16.5 (12.3, 20.4)	0.062	
M2	19.4 (12.9, 23.8)	17.5 (13.1, 24.1)	0.893		14.5 (12.2, 18.7)	0.127	
IL-12/23p40 (pg/mL)				0.932			0.135
M1	105 (86, 148)	110 (83, 164)	0.897		117 (90, 150)	0.500	

M2	117 (82, 152)	101 (87, 163)	0.893		98 (81, 145)	0.168	
IL-15 (pg/mL)				0.177			0.424
M1	3.0 (2.5, 3.3)	2.9 (2.4, 3.3)	0.878		3.0 (2.6, 3.3)	0.750	
M2	2.7 (2.3, 3.2)	2.9 (2.5, 3.5)	**0.048**		2.9 (2.6, 3.2)	0.216	
IL-16 (pg/mL)				0.228			0.798
M1	187 (161, 226)	202 (167, 209)	0.689		201 (161, 234)	0.953	
M2	161 (146, 229)	196 (176, 223)	0.305		173 (140, 214)	0.376	
IL-17A (pg/mL)				0.083			0.864
M1	2.3 (1.5, 3.1)	2.3 (1.4, 3.0)	0.768		2.4 (1.5, 3.5)	0.842	
M2	2.3 (1.6, 3.5)	2.6 (2.1, 4.1)	0.110		2.2 (1.5, 3.5)	0.787	
TNF-B (pg/mL)				0.689			0.312
M1	0.4 (0.1, 0.5)	0.3 (0.1, 0.5)	0.472		0.3 (0.1, 0.6)	0.293	
M2	0.1 (0.1, 0.4)	0.1 (0.1, 0.4)	0.938		0.1 (0.1, 0.3)	0.508	
**Anti-inflammatory panel**							
IL-1sRII (ng/mL)				0.323			0.649
M1	22.9 (19.8, 28.1)	21.4 (17.7, 26.4)	0.123		23.2 (19.3, 26.2)	0.079	
M2	24.1 (21.1, 27.2)	24.5 (19.9, 27.3)	> 0.999		25.0 (19.6, 28.6)	0.946	
**Pro-inflammatory panel**							
IFN-A (pg/mL)				0.291			0.926
M1	66 (55, 79)	66 (55, 79)	0.856		66 (55, 100)	0.328	
M2	66 (38, 79)	76 (50, 97)	0.258		76 (55, 79)	0.313	
IFN-G (pg/mL)				0.376			0.317
M1	4.7 (3.3, 6.6)	4.8 (3.1, 7.7)	0.859		5.1 (3.1, 7.4)	0.786	
M2	5.9 (4.6, 14.4)	4.7 (4.4, 18.7)	0.414		4.4 (3.4, 8.8)	0.244	
IL-2R (pg/mL)				0.300			0.633
M1	119 (95, 177)	109 (94, 177)	0.817		130 (95, 150)	0.812	
M2	153 (95, 229)	164 (125, 229)	0.233		116 (88, 218)	0.377	
IL-6 (pg/mL)				0.711			0.073
M1	0.6 (0.4, 0.8)	0.6 (0.4, 0.9)	0.804		0.6 (0.5, 0.8)	0.714	
M2	0.9 (0.6, 1.1)	0.8 (0.5, 1.7)	0.635		0.6 (0.5, 0.9)	0.057	
IL-8 (pg/mL)				0.360			0.568
M1	10.6 (8.4, 12.2)	10.3 (7.9, 12.9)	0.100		10.1 (7.6, 13.2)	0.400	
M2	9.6 (6.8, 15.5)	8.2 (6.4, 12.0)	0.110		8.3 (6.6, 10.2)	0.244	
TNF-A (pg/mL)				0.303			0.568
M1	1.9 (1.3, 2.2)	1.7 (1.4, 2.3)	0.258		1.8 (1.3, 2.3)	0.915	
M2	2.0 (1.3, 2.3)	2.1 (1.7, 2.1)	0.735		1.6 (1.3, 2.7)	0.542	
COMP (U/L)				0.544			0.938
M1	11.2 (8.9, 12.1)	10.1 (8.6, 12.4)	0.377		10.9 (8.4, 13.3)	0.542	
M2	10.8 (8.8, 13.7)	11.0 (9.7, 13.7)	0.946		10.1 (9.1, 13.8)	0.700	
**Angiogenesis panel**							
VEGF (pg/mL)[3]				0.168			0.955
M1, M1/2-1	134 (60, 181)	116 (56, 200)	0.279		97 (59, 204)	0.500	
M2, M1/2-2	93 (51, 228)	104 (64, 229)	0.273		108 (62, 226)	>0.999	
VEGF-A (pg/mL)				0.669			0.493
M1	299 (164, 470)	309 (134, 467)	0.427		266 (138, 436)	0.679	
M2	306 (98, 608)	215 (152, 465)	0.273		352 (109, 485)	0.735	
VEGF-C (pg/mL)				0.252			0.127
M1	395 (374, 523)	396 (334, 502)	0.194		389 (336, 514)	0.662	
M2	406 (334, 494)	479 (344, 529)	0.685		473 (341, 562)	**0.048**	
VEGF-D (ng/mL)				0.493			0.588
M1	0.7 (0.7, 0.9)	0.7 (0.6, 1.0)	0.470		0.8 (0.6, 0.9)	0.400	
M2	0.7 (0.6, 0.9)	0.6 (0.6, 0.9)	0.273		0.6 (0.6, 0.8)	0.080	
Tie-2 (ng/mL)				0.317			0.094
M1	4.5 (4.2, 5.3)	4.5 (3.7, 5.2)	0.239		4.7 (3.7, 4.9)	**0.028**	
M2	4.1 (3.9, 5.1)	4.5 (3.4, 5.5)	0.588		4.2 (3.8, 4.9)	0.839	
Flt-1 (pg/mL)				0.648			0.493
M1	88 (74, 99)	87 (76, 98)	0.455		89 (78, 112)	0.915	
M2	93 (78, 97)	83 (64, 122)	> 0.999		86 (77, 96)	0.191	
bFGF (pg/mL)				0.842			0.977
M1	4.7 (2.2, 6.8)	3.8 (2.8, 7.6)	0.628		4.3 (3.0, 9.2)	0.324	
M2	3.2 (1.2, 4.9)	2.9 (2.1, 3.9)	0.635		3.9 (2.1, 7.7)	0.216	
HGF-1 (pg/mL)				0.882			0.932
M1	154 (108, 235)	154 (86, 223)	0.392		171 (86, 223)	0.784	
M2	212 (86, 235)	166 (114, 295)	0.926		212 (94, 283)	>0.999	
**Vascular injury panel**							

hs-CRP (mg/L)				0.194			**0.029**
M1	0.9 (0.4, 1.5)	1.0 (.4, 2.3)	**0.049**		0.9 (0.5, 1.3)	0.714	
M2	1.2 (0.8, 3.0)	0.9 (0.7, 1.8)	0.700		0.7 (0.5, 2.0)	0.050	
ICAM-1 (μg/mL)				0.493			0.345
M1	0.4 (0.3, 0.5)	0.4 (0.3, 0.5)	0.878		0.3 (0.3, 0.5)	**0.045**	
M2	0.4 (0.4, 0.5)	0.4 (0.3, 0.5)	0.414		0.4 (0.3, 0.5)	0.305	
VCAM-1 (μg/mL)				0.608			0.808
M1	0.6 (0.5, 0.8)	0.6 (0.5, 0.7)	0.662		0.6 (0.5, 0.7)	0.136	
M2	0.6 (0.6, 0.7)	0.6 (0.4, 0.7)	0.455		0.6 (0.5, 0.7)	0.305	
SAA (μg/mL)				0.168			0.210
M1	1.6 (1.0, 2.2)	2.1 (1.3, 4.4)	**0.009**		1.5 (1.0, 3.1)	0.220	
M2	1.9 (0.9, 3.9)	1.9 (0.8, 3.9)	0.839		1.9 (1.1, 2.4)	0.542	

[1] Significance from Wilcoxon signed rank test; [2] Significance from Mann-Whitney's test. bFGF = Fibroblast growth factor beta, COMP = cartilage oligomeric matrix protein, CTX1 = C telopeptide of type I collagen, Flt = fms related tyrosine kinase (=VEGFR1), HGF = hepatocyte growth factor, hs-CRP = high-sensitive C-reactive protein, ICAM = intercellular adhesion molecule, IL = interleukin, IFN = interferon, IP = interferon-γ-inducible protein, iPINP = intact procollagen I N-terminal propeptide, MCP = Monocyte chemotactic protein, MDC = Macrophage derived chemokine, MIG = Monokine induced by gamma-interferon, MIP = macrophage inflammatory protein, RANKL = Receptor activator of NF-κB ligand, RANTES = Regulated upon activation, normally T-expressed, and presumably secreted, SAA = Serum amyloid A, TARC = thymus and activation-regulated chemokine, Tie = TEK receptor tyrosine kinase, TNF = tumor necrosis factor, VCAM = vascular cell adhesion molecule, VEGF = vascular endothelial growth factor.

Table S2. Spearman's correlation coefficients between one-year changes in total, Type 1 (M1) and Table 2. (M2) volumes of primary Modic changes (MC) and concentrations of serum biomarkers according to intervention: zoledronic acid (ZA) or placebo infusion. Significant changes bolded. Positive correlation values note higher biomarker concentration with higher volume change.

	**Change in Primary MC Total Volume**		**Change in Primary M1 Volume**		**Change in Primary M2 Volume**	
	**Placebo**	**ZA**	**Placebo**	**ZA**	**Placebo**	**ZA**
**Bone panel**						
AFOS	0.18	0.38	0.02	0.27	−0.01	−0.35
RANKL	0.07	−0.10	0.35	−0.26	−0.15	**0.48**
iPINP	0.21	**0.65**	0.06	**0.49**	0.09	−0.21
CTX-1	0.16	−0.03	0.10	−0.12	0.00	0.09
**Chemokine panel**						
Eotaxin-1	0.11	0.18	0.00	0.06	−0.04	−0.05
Eotaxin-3	0.28	0.06	0.07	−0.10	0.21	0.12
IP-10	0.16	0.02	−0.19	0.03	0.43	−0.17
MIP-1A	0.19	0.22	0.09	0.03	0.12	0.19
MIP-1B	−0.10	0.25	0.21	0.23	−0.19	−0.30
MCP-1	0.24	0.03	0.16	−0.07	0.08	0.10
MCP-4	0.20	0.20	0.04	0.23	−0.02	−0.19
MDC-1	0.18	0.19	−0.02	0.05	0.02	0.05
RANTES	−0.12	0.10	0.18	0.22	−0.37	−0.02
TARC	0.17	0.15	0.02	0.11	−0.04	−0.01
MIG-1	**0.58**	0.20	**0.48**	0.15	0.18	−0.20
**Cytokine panel**						
IL-7	0.20	0.00	0.22	−0.21	0.04	**0.46**
IL-12/23p40	0.10	0.16	**0.47**	0.05	−0.43	−0.04
IL-15	0.22	0.02	0.43	−0.10	−0.25	0.06
IL-16	0.09	**0.53**	0.13	0.23	−0.13	−0.07
IL-17A	−0.02	−0.07	−0.24	−0.27	−0.06	0.36
TNF-B	−0.09	−0.11	0.29	−0.23	−0.36	0.26
**Anti-inflammatory panel**						
IL-1sRII	−0.19	−0.29	−0.12	−0.13	−0.05	−0.27
**Pro-inflammatory panel**						

IFN-A	0.33	0.02	0.43	0.00	−0.11	−0.11
IFN-G	0.05	0.21	−0.20	0.11	0.20	−0.09
IL-2R	0.09	0.43	−0.26	0.35	0.23	−0.27
IL-6	−0.08	0.13	−0.28	0.00	0.26	0.00
IL-8	0.22	−0.07	0.05	−0.44	0.10	**0.66**
TNF-A	−0.11	−0.02	−0.32	0.04	0.02	−0.15
**Angiogenesis panel**						
VEGF-A	0.34	0.30	0.04	0.07	**0.45**	0.20
VEGF-C	0.17	0.13	0.30	−0.09	−0.12	0.14
VEGF-D	0.14	0.35	0.03	−0.12	0.19	0.43
Tie-2	**0.51**	−0.20	**0.48**	−0.10	0.11	−0.09
Flt-1	0.29	0.00	0.22	−0.27	0.20	**0.47**
bFGF	0.00	−0.18	−0.13	0.09	0.22	−0.30
HGF-1	0.09	−0.02	**0.47**	−0.03	−0.37	0.00
**Vascular injury panel**						
hs-CRP	0.10	0.25	−0.23	0.12	0.42	−0.08
ICAM-1	0.29	−0.03	0.30	0.11	0.07	−0.14
VCAM-1	0.15	−0.11	0.17	0.05	0.10	−0.12
SAA	−0.13	0.00	−0.37	0.03	0.28	−0.21

AFOS = alkaline phosphatase, bFGF = basic fibroblast growth factor beta, CTX1 = C telopeptide of type I collagen, Flt = fms related tyrosine kinase (=VEGFR1), HGF = hepatocyte growth factor, hs-CRP = high-sensitive C-reactive protein, ICAM = intercellular adhesion molecule, IL = interleukin, IFN = interferon, IP = interferon-γ-inducible protein, iPINP = intact procollagen I N-terminal propeptide, MCP = monocyte chemotactic protein, MDC = macrophage derived chemokine, MIG = monokine induced by gamma-interferon, MIP = macrophage inflammatory protein, RANKL = receptor activator of NF-κB ligand, RANTES = regulated upon activation, normally T-expressed, and presumably secreted, SAA = serum amyloid A, TARC = thymus and activation-regulated chemokine, Tie = TEK receptor tyrosine kinase, TNF = tumor necrosis factor, VCAM = vascular cell adhesion molecule, VEGF = vascular endothelial growth factor.

Table S3. Spearman's correlation coefficients between change in low back pain intensity (VAS) at one month and one year and concentrations of serum biomarkers according to intervention: zoledronic acid (ZA) or placebo infusion. Significant changes bolded. Positive correlation values note higher biomarker concentration with higher LBP intensity (VAS).

	Change in LBP (VAS) 1 Month		Change in LBP (VAS) 1 Year	
	**Placebo**	**ZA**	**Placebo**	**ZA**
**Bone panel**				
AFOS	−0.36	0.16	−0.03	−0.39
RANKL	−0.33	0.14	0.25	−0.22
iPINP	−0.01	0.12	−0.01	−0.10
CTX-1	0.33	0.29	−0.41	0.01
**Chemokines panel**				
Eotaxin-1	0.52	0.08	0.26	0.01
Eotaxin-3	0.33	0.43	0.24	0.04
IP-10	0.02	0.25	−0.27	0.04
MIP-1A	−0.28	−0.49	−0.06	−0.02
MIP-1B	−0.23	0.34	0.15	0.30
MCP-1	0.10	0.04	0.32	0.13
MCP-4	0.45	0.07	0.41	0.18
MDC-1	0.10	0.47	0.24	−0.06
RANTES	0.12	0.55	0.01	−0.01
TARC	0.15	0.42	0.26	0.33

MIG-1	−0.14	0.18	−0.20	−0.11
**Cytokine panel**				
IL-7	0.03	0.01	−0.20	−0.14
IL-12/23p40	0.08	0.39	−0.38	0.07
IL-15	0.11	0.15	0.06	0.03
IL-16	0.19	0.19	0.00	−0.09
IL-17A	−0.01	0.29	−0.04	−0.07
TNF-B	0.09	0.45	0.08	0.13
**Anti-inflammatory panel**				
IL-1sRII	−0.08	0.28	0.06	0.04
**Pro-inflammatory panel**				
IFN-A	0.20	−0.18	−0.08	−0.13
IFN-G	−0.31	−0.24	0.28	−0.22
IL-2R	−0.28	0.26	0.00	−0.22
IL-6	−0.08	0.09	−0.03	−0.36
IL-8	0.07	0.26	0.10	0.07
TNF-A	−0.27	0.01	−0.04	−0.07
**Angiogenesis panel**				
VEGF-A	−0.21	0.07	0.07	−0.19
VEGF-C	0.22	0.41	0.37	0.09
VEGF-D	−0.24	−0.12	−0.22	−0.45
Tie-2	0.37	0.25	0.29	−0.12
Flt-1	−0.33	0.32	−0.15	−0.31
bFGF	0.00	0.01	0.23	0.22
HGF-1	0.12	0.03	0.22	0.00
**Vascular injury panel**				
hs-CRP	−0.12	−0.05	−0.49	−0.22
ICAM-1	0.03	−0.06	0.26	0.16
VCAM-1	−0.07	0.04	0.32	0.27
SAA	−0.01	0.08	−0.25	−0.12

AFOS = alkaline phosphatase, bFGF = basic fibroblast growth factor beta, CTX1 = C telopeptide of type I collagen, Flt =fms related tyrosine kinase (=VEGFR1), HGF = hepatocyte growth factor, hs-CRP = high-sensitive C-reactive protein, ICAM = intercellular adhesion molecule, IL = interleukin, IFN = interferon, IP = interferon-γ-inducible protein, iPINP = intact procollagen I N-terminal propeptide, MCP = monocyte chemotactic protein, MDC = macrophage derived chemokine, MIG = monokine induced by gamma-interferon, MIP = macrophage inflammatory protein, RANKL = receptor activator of NF-κB ligand, RANTES = regulated upon activation, normally T-expressed, and presumably secreted, SAA = serum amyloid A, TARC = thymus and activation-regulated chemokine, Tie = TEK receptor tyrosine kinase, TNF = tumor necrosis factor, VCAM = vascular cell adhesion molecule, VEGF = vascular endothelial growth factor.
