# Supplementary material for: The Effect of Zoledronic Acid on Serum Biomarkers among Patients with Chronic Low Back Pain and Modic Changes in Lumbar Magnetic Resonance Imaging
Source: Diagnostics (Basel). 2019 Dec 4;9(4):212. doi: 10.3390/diagnostics9040212 (PMC6963270; doi:10.3390/diagnostics9040212)
Supplement: Supplementary file 1 [file diagnostics-09-00212-s001.zip › Suppl. figures.pdf]

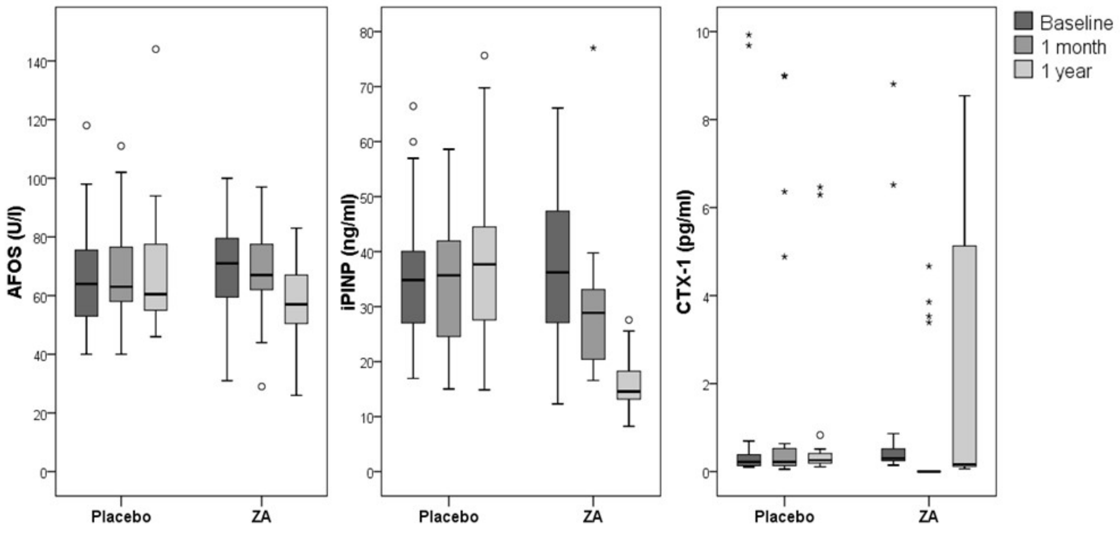

Figure S1. The levels of alkaline phosphatase (AFOS), intact procollagen I N-terminal propeptide (iPINP) and C telopeptide of type I collagen (CTX-1) at baseline, one-month and one-year follow-up in the zoledronic acid (ZA, *n* = 20) or placebo (*n* = 20) infusion groups. The data are shown by box plot, median and IQR ± min/max. At the one-month follow-up iPINP had decreased in the ZA group. The difference between the placebo and ZA groups from baseline to one month was significant for iPINP and CTX-1. At the one-year follow-up AFOS and iPINP had decreased in the ZA group.

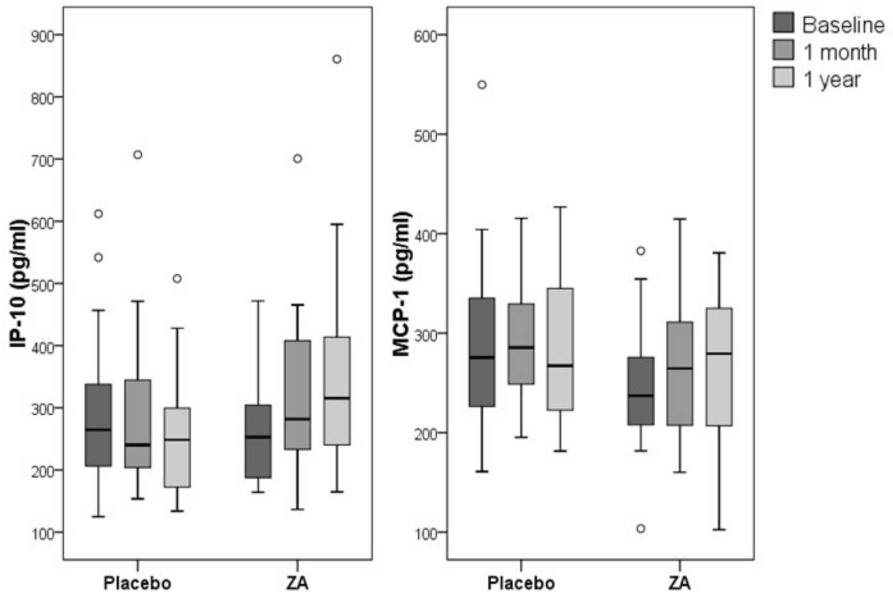

Figure S2. The levels of interferon-γ-inducible protein (IP)-10 and monocyte chemotactic protein (MCP)-1 in the zoledronic acid (ZA, *n* = 20) or placebo (*n* = 20) infusion groups. The data are shown by box plot, median and IQR ± min/max. At the one-month follow-up IP-10 and MCP-1 had elevated in the ZA group. At the one-year follow-up IP-10 had elevated in the ZA group. During the one-year follow-up IP-10 had decreased in the placebo group and elevated in the ZA group.

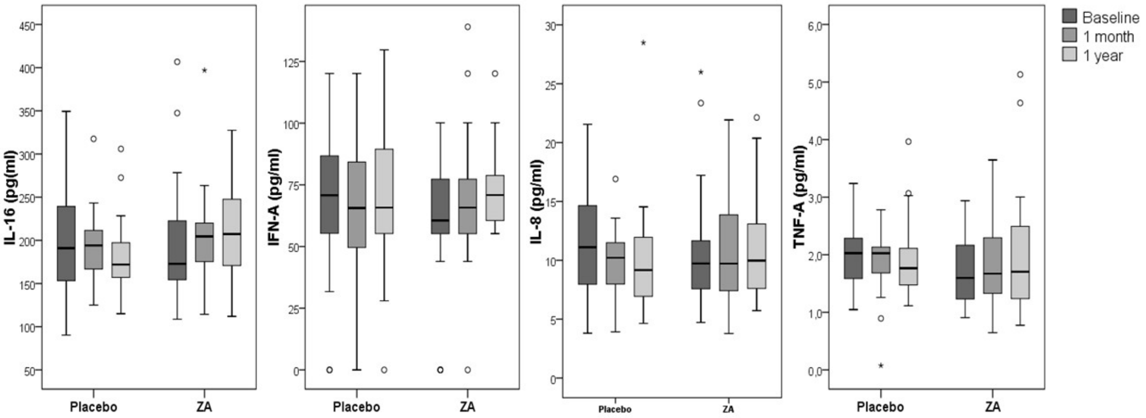

Figure S3. The levels of interleukin (IL)-16, interferon (IFN)-A, IL-8 and tumour necrosis factor (TNF)-A in the zoledronic acid (ZA, *n* = 20) or placebo (*n* = 20) infusion groups. The data are shown by box plot, median and IQR ± min/max. IL-16 in the placebo group had decreased at the one-year follow-up, while IFN-A had elevated in the ZA group. During the one-year follow-up IL-16 and TNF-A had decreased in the placebo group and elevated in the ZA group.

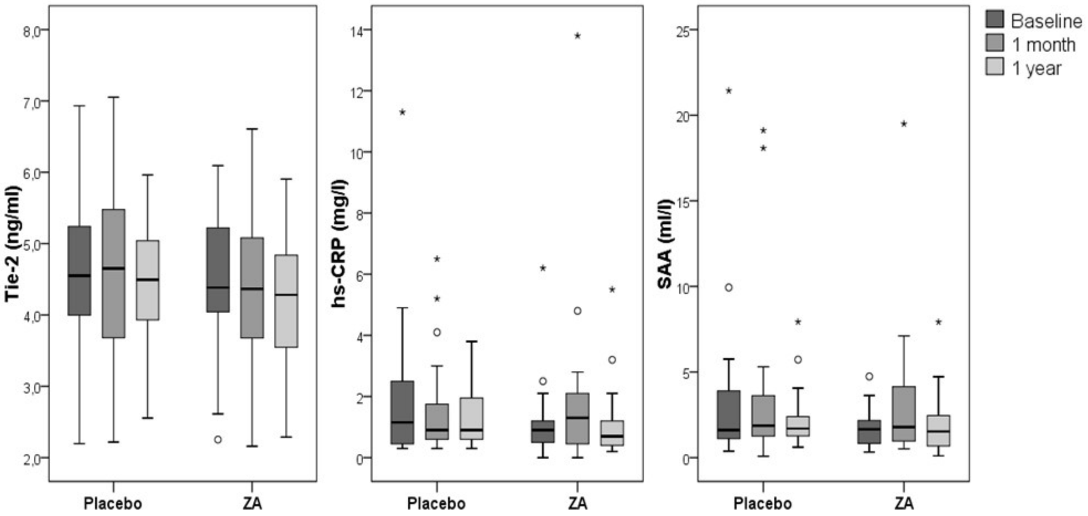

Figure S4. The levels of TEK receptor tyrosine kinase (Tie)-2, high-sensitive C-reactive protein (hs-CRP) and serum amyloid A (SAA) in the zoledronic acid (ZA, *n* = 20) or placebo (*n* = 20) infusion groups. The data are shown by box plot, median and IQR ± min/max. At the one-month follow-up SAA and hs-CRP had elevated in the ZA group. At the one-year follow-up Tie-2 had decreased in the ZA group.
